# Identification of a robust bacterial pyranose oxidase that displays an unusual pH dependence

**DOI:** 10.1016/j.jbc.2024.107885

**Published:** 2024-10-11

**Authors:** Lars L. Santema, Henriëtte J. Rozeboom, Veronica P. Borger, Saniye G. Kaya, Marco W. Fraaije

**Affiliations:** Molecular Enzymology, University of Groningen, Groningen, The Netherlands

**Keywords:** pyranose oxidase, bacterial pyranose oxidase, covalent flavin, monosaccharides, glucose

## Abstract

Pyranose oxidases are valuable biocatalysts, yet only a handful of bacterial pyranose oxidases are known. These bacterial enzymes exhibit noteworthy distinctions from their extensively characterized fungal counterparts, encompassing variations in substrate specificity and structural attributes. Herein a bacterial pyranose oxidase from *Oscillatoria princeps* (*O*POx) was biochemically characterized in detail. In contrast to the fungal pyranose oxidases, *O*POx could be well expressed in *Escherichia coli* as soluble, fully flavinylated, and active oxidase. It was found to be highly thermostable (melting temperature >90 °C) and showed activity on glucose, exhibiting an exceptionally low *K*_*M*_ value (48 μM). Elucidation of its crystal structure revealed similarities with fungal pyranose oxidases, such as being a tetramer with a large central void leading to a narrow substrate access tunnel. In the active site, the FAD cofactor is covalently bound to a histidine. *O*POx displays a relatively narrow pH optimum for activity with a sharp decline at relatively basic pH values which is accompanied by a drastic change in its flavin absorbance spectrum. The pH-dependent switch in flavin absorbance features and oxidase activity was shown to be fully reversible. It is hypothesized that a glutamic acid helps to stabilize the protonated form of the histidine that is tethered to the FAD. *O*POx presents itself as a valuable biocatalyst as it is highly robust, well-expressed in *E. coli*, shows low *K*_*M*_ values for monosaccharides, and has a peculiar pH-dependent “on-off switch”.

Carbohydrate oxidases are an ever-expanding class of enzymes of considerable industrial significance. By utilizing a redox cofactor they perform oxidations of carbohydrates under mild conditions with high regioselectivity (1). Carbohydrate oxidases can be divided into three families based on their use of cofactor and specific structural fold. One of which is the glucose-methanol-choline (GMC) family in which FAD is the crucial flavin redox cofactor (2). The mechanism of GMC-type flavoprotein oxidases can be divided into two half-reactions. First, in the so-called reductive half-reaction, the substrate is oxidized by hydride transfer to the flavin cofactor. This part of the mechanism determines the regioselectivity of oxidation. Next, in the so-called oxidative half-reaction, the reduced flavin cofactor is reoxidized by donating two electrons to molecular oxygen resulting in the production of hydrogen peroxide (3). Being merely dependent on dioxygen for activity, a large number of GMC-type oxidases have found applications across diverse industries (4, 5, 6, 7, 8).

Perhaps the most extensively explored GMC-type oxidases are those acting on glucose and other saccharides. Glucose oxidase from *Aspergillus niger* (*An*GOx) is the most studied and used carbohydrate oxidase and can be found in numerous applications (9). It efficiently oxidizes D-glucose at the C1 position to form gluconic acid. Another well-studied but less employed class of GMC-type monosaccharide oxidases is the class of pyranose oxidases (POx) which typically oxidize monosaccharides at the C2 position. Based on sequence similarity and the glucose-2 oxidase activity, POxs have been grouped as members of CAZY Auxiliary Activity Family 3, subfamily 4 (AA3 4) (10). POxs are typically found in wood-degrading fungal species (11). They are thought to fuel lignin degradation by the production of hydrogen peroxide that is required by other oxidative enzymes (peroxidases and peroxygenases) that attack the lignin polymer. They are also considered for biocatalytic applications. For example, they can be employed to produce intermediates for the antibiotic cortalcerone or help to reduce radicals and various quinones (12, 13, 14). Fungal POxs have been extensively studied and their crystal structures have revealed that they form homotetramers with a big central void from where narrow substrate access tunnels lead to the active site. In these flavoenzymes, the flavin cofactor is covalently bound *via* a histidine (14). They typically exhibit a strict C2 regioselectivity, convert both anomers, and show a relatively high affinity for monosaccharides (15, 16, 17). Regardless of these favorable characteristics, the industrial use of POxs appears to be less developed when compared with GOxs (15).

One of the reasons for relatively few POx-based applications may be the difficulty of recombinantly expressing these fungal enzymes in *Escherichia coli*. A solution to this may be the exploitation of bacterial homologs. Yet, so far, merely a handful of bacterial POxs have been biochemically characterized (18, 19, 20, 21). This has revealed that bacterial POxs often behave very differently from their fungal counterparts. In contrast to fungal POxs, bacterial POxs are often monomeric or dimeric and rarely contain a covalently bound flavin cofactor (15). Their physiological function has been proposed also to be linked to lignin degradation (22). However, many of the described bacterial POxs do not show activity on simple monosaccharides such as glucose but are specific for glycosides thereby acting as *C*-glycoside-3-oxidases (G3Ox) rather than pyranose oxidases (23). All in all, access to an easy-to-express POx able to efficiently oxidize glucose is still highly desirable.

Herein, a POx from the cyanobacterium *Oscillatoria princeps* (*O*POx) is described as a bona vide bacterial homolog to the fungal POxs as its biochemical characterization and structural elucidation show many properties shared with its fungal counterparts such as substrate profile and regioselectivity. Furthermore, it exhibits other unique features that are interesting in view of biocatalytic applications.

## Results

### Identification of the bacterial pyranose oxidase homolog

Utilizing the sequence of the fungal POx from *Trametes ochracea* (*To*POx) (GenBank: 1TT0_A) as a search query, a PSI-BLAST search in sequenced bacterial genomes was performed (Table S1). By sequence alignment and structural comparison of predicted AlphaFold models (24), the putative POx from the cyanobacterium *O. princeps* (GenBank: MBW4497712.1, 534 amino acids) dubbed *O*POx was identified. The putative POx exhibits a notable resemblance to *To*POx, with a sequence identity of 35% and a root mean square deviation (RMSD) of 0.7 Å between its predicted structure and the *To*POx crystal structure (PDB: 1TT0). The model of *O*POx predicts the presence of a ‘head’ and an ‘arm’ domain which are thus far rarely observed in bacterial POxs and are believed to be important for oligomerization (15, 25). A multiple sequence alignment between the biochemically characterized POxs (Fig. S1) suggests that *O*POx contains the conserved catalytic histidine and asparagine, the covalent FAD binding histidine and the threonine which is believed to stabilize the C4a intermediate in the oxidative half-reaction of *To*POx (26, 27). A DALI structural search (28) of the ColabFold (29) predicted structure showed, besides high similarity with *To*POx, also a low RMSD value (1.6 Å) with the newly discovered G3Ox from *Pseudarthrobacter siccitolerans* (*Ps*G3Ox) (30). Nevertheless, the *O*POx model shows a clear arm domain whereas *Ps*G3Ox and other G3Oxs have an insertion-1 segment, which protects the active site against solvents and is the distinctive structural difference between POxs and G3Oxs (30). These findings hint towards *O*POx being a true pyranose 2-oxidase and not a glycoside-3-oxidase. In sum, the observations above suggest that *O*POx is a bacterial POx with certain features conventionally associated with fungal POxs which prompted our detailed biochemical study on *O*POx.

### Expression, purification, absorbance spectra, and hydrodynamic properties

Soluble recombinant *O*POx was obtained by cloning a synthetic gene encoding *O*POx in a pBAD vector, resulting in the expression of the enzyme with an N-terminal His-tag by utilizing *E. coli* NEB 10-beta as an expression host. A two-step purification process consisting of an immobilized metal affinity chromatography and a heat purification step resulted in 17 mg of pure yellow-colored protein per liter of culture. SDS-PAGE analysis (Fig. S2*A*) showed a single protein band at ∼65 kDa, which is in line with the predicted mass of the protein with a His-tag (61 kDa). Incubating the polyacrylamide gel in acetic acid (5% v/v) revealed the same band under UV-light exposure revealing that the flavin cofactor is covalently bound (31). Only one other bacterial POx was previously shown to contain a covalently bound FAD cofactor (18). A UV-vis absorbance spectrum collected at pH 5.5 showed two characteristic flavin absorbance peaks at 360 nm and 455 nm. The ratio in absorbance at 280 nm and 455 nm was found to be 11.7 which suggests that *O*POx is mainly obtained in its holo form. By collecting the flavin absorbance spectra of the native protein and that of the protein sample after unfolding by 0.1% SDS, the molar extinction coefficient could be determined for quantifying the amount of enzyme (ε_455nm_ = 13.1 mM^-1^ cm^-1^) (Fig. S2*B*).

**Figure 1:**
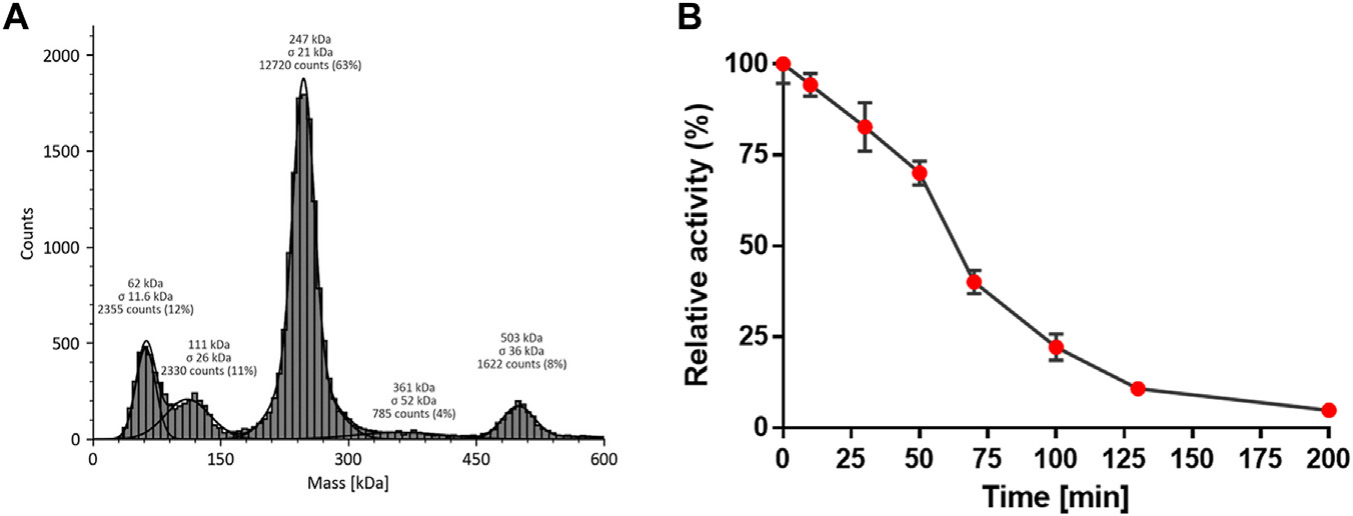
**Properties of*****O*****POx.***A*, mass photometry of 10 nM *O*POx in 50 mM citrate buffer (pH 5.5). *B*, half-life of *O*POx at 90 °C in 50 mM citrate buffer (pH 5.5). Remaining activity was measured using 1.0 mM glucose in the HRP-coupled assay at 25 °C. Error bars indicate SD and all data were measured in duplicates (*n* = 2).

By DLS and gel permeation chromatography experiments, it was found that *O*POx forms a tetramer in solution (Fig. S3). Mass photometry experiments revealed that even at low enzyme concentrations (10 nM), the enzyme mainly forms tetramers, while also some monomers, dimers, and octamers are observed (Fig. 1*A*). A tetrameric state has, until now, been exclusively identified for fungal POxs. This observation lends additional support to the hypothesis that the 'head' and 'arm' domains contribute to oligomerization, given their absence in most monomeric and dimeric bacterial POxs while being present in *O*POx (15, 25).

### Biochemical characterization

The thermostability of *O*POx was probed using the ThermoFluor assay (32), unveiling relatively high apparent melting temperatures, reaching 95 °C at pH 5, and a preference for slightly acidic conditions (Table 1). The enzyme appears to be resilient against common cosolvents such as DMSO and ethanol with apparent melting temperatures of >75 °C in the presence of 10% cosolvent. The thermal stability is also observed when measuring its half-life of inactivation: the enzyme retains 50% of its activity after being incubated at 90 °C for an hour (Fig. 1*B*). This establishes *O*POx as the most thermostable POx described to date, surpassing the thermostability of the most resilient bacterial POx (from *Kitasatospora aureofaciens*: 56 °C) and the most resilient fungal POx (from *Aspergillus oryza*: 75 °C) (18, 33).

**Table 1:** The apparent melting temperature of OPOx in various buffers and conditions

Buffer (50 mM)	Apparent melting temperature (°C)
Citrate buffer pH 4	85
Citrate buffer pH 4.5	91
Citrate buffer pH 5	95
Citrate buffer pH 5.5	93
Citrate buffer pH 6	90
KP_i_ buffer pH 6.5	90
KP_i_ buffer pH 7	85
KP_i_ buffer pH 7.5	82
10% DMSO, citrate buffer pH 5.5	87
10% Ethanol, citrate buffer pH 5.5	77
10% Methanol, citrate buffer pH 5.5	83
10% Glycerol, citrate buffer pH 5.5	91

The substrate scope of *O*POx was probed in a 96-well plate with various carbohydrates using a peroxidase-coupled assay (34) (Table S2). Like other biochemically characterized POxs, *O*POx is primarily active on monosaccharides, both *L*- and *D*-isomers. In contrast to the POx from *Streptomyces canus* and *Ps*G3Ox (19, 30), no activity on the tested glycosylated carbohydrates, carminic acid, and naringin, was observed.

To probe the regioselectivity and thus the product formed by the action of *O*POx, a conversion of D-glucose was analyzed using COSY NMR (Fig. S4). The NMR spectra show the disappearance of the interaction peak between the H1 and H2 group of both α- and β-glucose, indicating the oxidation site to be at the C2 of glucose and confirming the reactivity of the enzyme with both anomers. All-in-all, the results show that *O*POx is a true pyranose-2-oxidase, resembling the fungal POxs.

### Steady-state kinetics

The steady-state kinetic parameters for several substrates were determined in 50 mM citrate buffer (pH 5.5) at 25 °C using an HRP-based assay (34) (Table 2) (Fig. S5). The results reveal that *D*-glucose is the preferred substrate as it shows the highest catalytic efficiency (64 mM^-1^ s^-1^), followed by *D*-xylose (16 mM^-1^ s^-1^) and *D*-galactose (4.1 mM^-1^ s^-1^). These three monosaccharides are often described as preferential substrates of POxs (15). *O*POx has a remarkably low *K*_*M*_ for its substrates, with a *K*_*M*_ value of only 48 μM for *D*-glucose. To our knowledge, this is the lowest *K*_*M*_, so far described, for any oxidase active in *D*-glucose. It is ∼10-fold lower than the lowest reported *K*_*M*_ for D-glucose of a POx (*To*POx: 740 μM) (14) and ∼500-fold lower than the *K*_*M*_ of the well-known and widely applied *An*GOx (22 mM) (35).

**Table 2:** Steady-state kinetic parameters of *O*POx in 50 mM citrate buffer (pH 5.5)

Substrate	*K*_*M*_ (mM)	*k*_*cat*_ (s^-1^)	*k*_*cat*_*/K*_*M*_ (mM^-1^/s^-1^)
*D*-glucose	0.048 ± 0.010	3.1 ± 0.3	64
*D-*xylose	0.16 ± 0.02	2.6 ± 0.1	16
*D-*galactose	0.68 ± 0.15	2.8 ± 0.2	4.1
*D*-galacturonic Acid	11 ± 3	1.0 ± 0.1	93 × 10^-3^
*D*-rhamnose	149 ± 28	3.7 ± 0.2	25 × 10^-3^
*D*-ribose	159 ± 35	1.6 ± 0.2	10 × 10^-3^
*L*-fucose	212 ± 14	2.1 ± 0.1	99 × 10^-4^

Rates were measured using an HRP-based method and performed in triplicates. Molecular oxygen was the electron acceptor and the experiments were performed at 25 °C.

The *K*_*M*_ for oxygen was probed by following the consumption of oxygen by *O*POx using an excess of D-glucose, revealing a relatively high *K*_*M*_ value of 131 μM for dioxygen (Fig. S6). This is in line with observations for other POxs and hints that, like other POxs, *O*POx can also utilize alternative electron acceptors (14, 15, 18).

### Pre-steady-state kinetics and redox potentials

**Figure 2:**
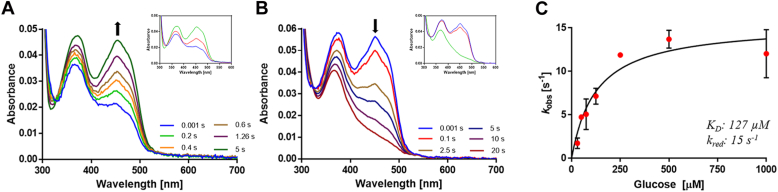
**Absorbance spectra and kinetics of*****O*****POx.***A*, selected flavin absorbance spectra upon mixing (1:1) 8.0 μM D-glucose-reduced *O*POx with aerobic buffer (∼240 μM oxygen) are shown, while the inset shows deconvoluted spectra. *B*, selected flavin absorbance spectra upon anaerobic mixing of 8 μM *O*POx with 500 μM D-glucose are shown, while the inset shows deconvoluted spectra. *C*, plot of *O*POx reduction rates at different concentrations of D-glucose. All kinetic analysis experiments were performed in 50 mM citrate buffer, pH 5.5. Error bars indicate SD and all points were taken in triplicates (*n* = 3).

The closely homologous *To*POx shows a peculiar pH dependency, where the oxidative half-life switched between a bi-bi ping-pong mechanism at pH values below 8, with an observable C4a-hydroperoxyflavin intermediate, and a bi-bi ordered mechanism at higher pH values (36, 37). The C4a-hydroperoxyflavin is stabilized by a conserved threonine (T127 in *O*POx) of which the Oγ of the hydroxyl group interacts with the N5 of the flavin cofactor (27). It has even been suggested that the decay of this C4a-hydroperoxyflavin is the rate-limiting step in the turnover of D-glucose by *To*POx (38). To investigate if *O*POx also forms and stabilizes this C4a-hydroperoxyflavin intermediate, anaerobically glucose-reduced *O*POx was mixed with aerobic buffer at pH 5.5 using a stopped-flow apparatus. Deconvolution of the spectra data revealed a two-phase process with a difference of ∼10 fold in their respective reoxidation rates while each process seems to reflect a reoxidation of the fully reduced flavin into fully oxidized flavin (Fig. 2*A*). These kinetic features hint to two different populations of the enzyme with roughly equal concentrations. No formation of a C4a-hydroperoxyflavin intermediate could be observed. The quicker phase occurred at a reoxidation rate of 3.0 s^-1^ when 0.12 mM molecular oxygen was used. Two kinetic events were also observed when monitoring the anaerobic reduction of *O*POx with D-glucose. Again, both processes reflect a transition from fully oxidized to fully reduced flavin (Fig. 2*B*) with about 10% of the flavin reduction occurring at a rate of 12 s^-1^ and 90% of the apparent reduction was relatively slow, 0.2 s^-1^. The reduction rate was found to be dependent on substrate concentration with a maximum rate of reduction (*k*_*red*_) of 15 s^-1^ and a K_d_ of 127 μM (Fig. 2*C*).

Based on sequence homology, it was inferred that His125 would form the covalent FAD-protein bond. To verify this and to reveal the effect of breaking the flavin-protein linkage, a mutant *O*POx was prepared in which the His125 was replaced with an Ala (H125A *O*POx). The mutant indeed did not form a covalent flavin-protein bond as the protein did not retain fluorescence upon SDS-PAGE analysis (Fig. S2*A*). The ratio in absorbance at 280 nm and 455 nm was found to be 13 which suggests that, like the wild-type enzyme, the purified H125A mutant is also mainly in its holo form. The H125A mutant also displayed a 10-fold reduced *k*_*cat*_ (0.3 s^-1^) and a 7-fold increased *K*_*M*_ value (350 μM) (Fig. S5*H*). Clearly, the covalent cofactor-protein tethering is beneficial for catalysis. Such an effect has been observed before in other flavoprotein oxidases and has been attributed to the relatively high redox potential of histidyl-linked flavins (39). To investigate this, redox titrations were performed. The redox potential of wild-type *O*POx was determined using the xanthine/xanthine oxidase method with indigotetrasulfonate (E_0_ = −46 mV) as reference dye (40, 41). This revealed a relatively high redox potential of −28 mV. The redox potential of H125A *O*POx was found to be lower (−44 mV, Fig. S7), in line with a lower oxidase activity. This confirms that the covalent tethering has an effect on the redox potential as has been observed for other covalent flavoproteins (39). The redox potential of *O*POx is somewhat higher when compared with other POxs, both from fungal and bacterial sources (15). Yet, other redox potentials were measured at different pH values and other buffer types, which influences redox properties. All-in-all, the results suggest that the covalent flavin-protein linkage has a similar influence on oxidase activity as described for *To*POx and other GMC oxidases (42, 43).

### Structural elucidation

**Figure 3:**
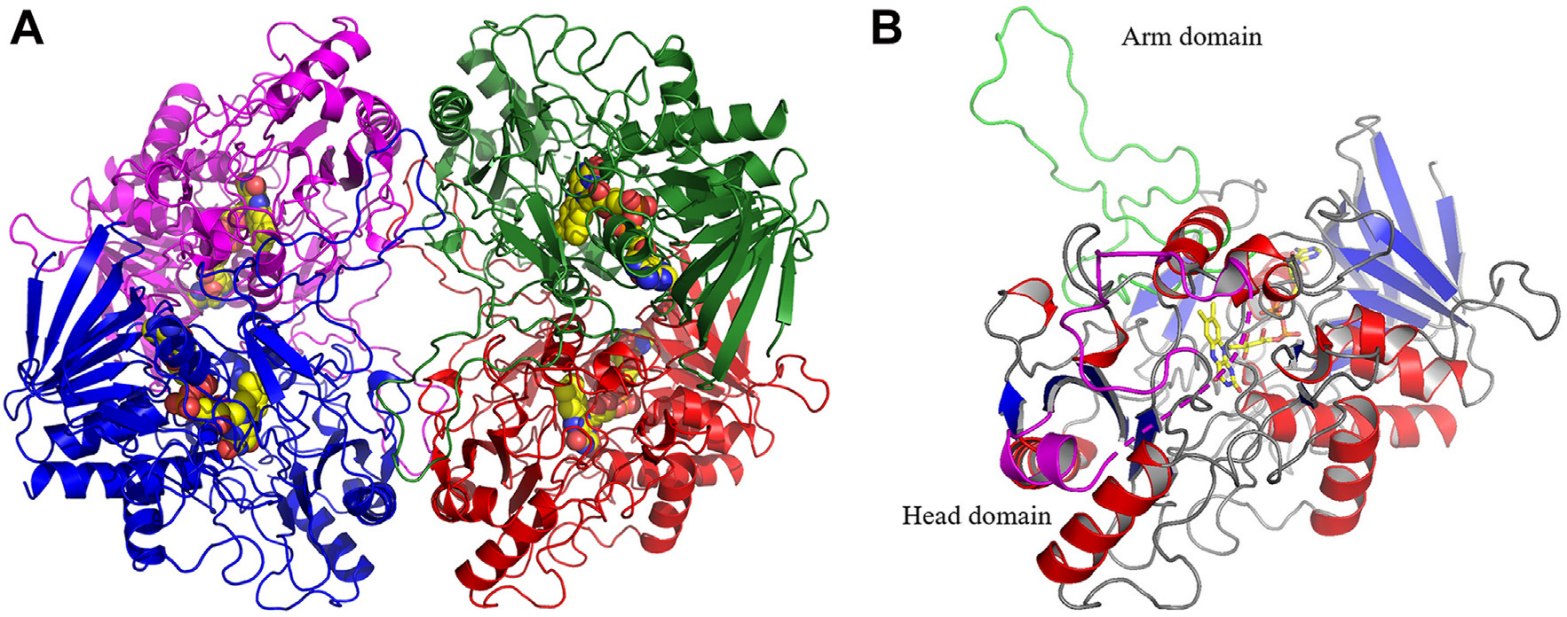
**Crystal structure of*****O*****POx.***A*, the four monomers are in different colors and FAD is shown as yellow spheres. *B*, monomer of *O*POx, α-helices are colored red and β-strands blue. The arm domain is shown in green, the head domain is shown in pink, and the flavin cofactor, in sticks, in cpk coloring.

Crystals of *O*POx could be obtained which allowed for the elucidation of its crystal structure. The crystal structure of *O*POx with a covalently bound FAD cofactor per subunit could be determined at 2.64 Å resolution in the orthorhombic space group *P*2_1_2_1_2_1_ with a tetrameric molecule in the asymmetric unit (Fig. 3*A*, PDB: 9FL2). According to the Pisa web server (44) the interface area in the crystal structure between subunits A-B (C-D) is 4000 Å^2^, and A-D (B-C) is 785 Å^2^, while between A-C (B-D) it is 685 Å^2^ with a Complexation Significance Score (CSS) score of 0.63. These values are the same for the low-resolution structure in space group C222_1_ although this crystal form contains a dimer in the asymmetric unit. Interfaces are formed by salt bridges, hydrogen bonds and hydrophobic interactions. In total, an area of 28,070 Å^2^ is buried upon tetramerization. A DALI search (28) highlighted its similarity with fungal POxs as it showed high homology (Zscores ∼49, ∼38–40% identical on residue level, RMSD of 1.3–2.0 Å) with *To*POx (633 residues, PDB: 1TT0) and other fungal POxs from *Peniophora* sp. (*Ps*POx, 595 residues, PDB: 1TZL) and *Phanerodontia chrysosporium* (*Pc*POx, 620 residues, PDB: 4MIF). Notably, *O*POx is shorter in sequence length than *To*POx (533 vs 633 residues). Some of these extra residues can be found in the head domain (protrusion) where they form a β-sheet and an α-helix. The head domain of *O*POx (residues 312–349) is significantly smaller than its fungal counterparts, 36 residues vs 61 residues in *To*POx and more similar in sequence size with the model of the dimeric bacterial POx from *K. aureofaciens* (38 residues) (18). In fungal POxs the head domain appears to be involved in oligomerization (25). However, no extensive interactions are observed to another subunit. Instead, the small *O*POx head domain has dimer contacts through residues His336 to Asp346 mainly by H-bonds which are absent in the fungal POxs. This is in contrast with a recent study that proposed the gradual extension of the head domain to be a major player in the oligomerization of POxs during the evolution in the clade from actinobacteria (23). It is noteworthy that *O*POx originates from a cyanobacterium rather than an actinobacterium. Nevertheless, the relatively small head domain and limited dimer interactions in *O*POx downplays the structural feature’s role in the oligomerization of the enzyme. Furthermore, *O*POx is missing 12 residues inserted between Arg292 and Pro293 (*O*POx numbering), and nine residues between Ile143 and Thr144 in comparison with *To*POx which are all inserts located on the surface of the tetramer. The arm domain (dimer loop) of *O*POx (48 residues, 69–116) seems to be of a similar size as its fungal counterparts. Also, structural homology is observed to G3Oxs (Zscores ∼17, ∼33% identical on residue level, RMSD of 1.6 Å) from *Microbacterium trichothecenolyticum* (PDB: 7DVE, 528 residues) and *Ps*G3Ox (PDB: 7QF8, 519 residues). In G3Oxs an insertion-1 segment is located between residues 60 to 93 which is involved in FAD incorporation, proper stabilization at the active site and plays a crucial role in catalysis (30). No insertion-1 segment is observed in *O*POx but rather at this location two small α-helices are observed which is similarly observed in fungal POxs (residues 54–67 in *O*POx).

**Figure 4:**
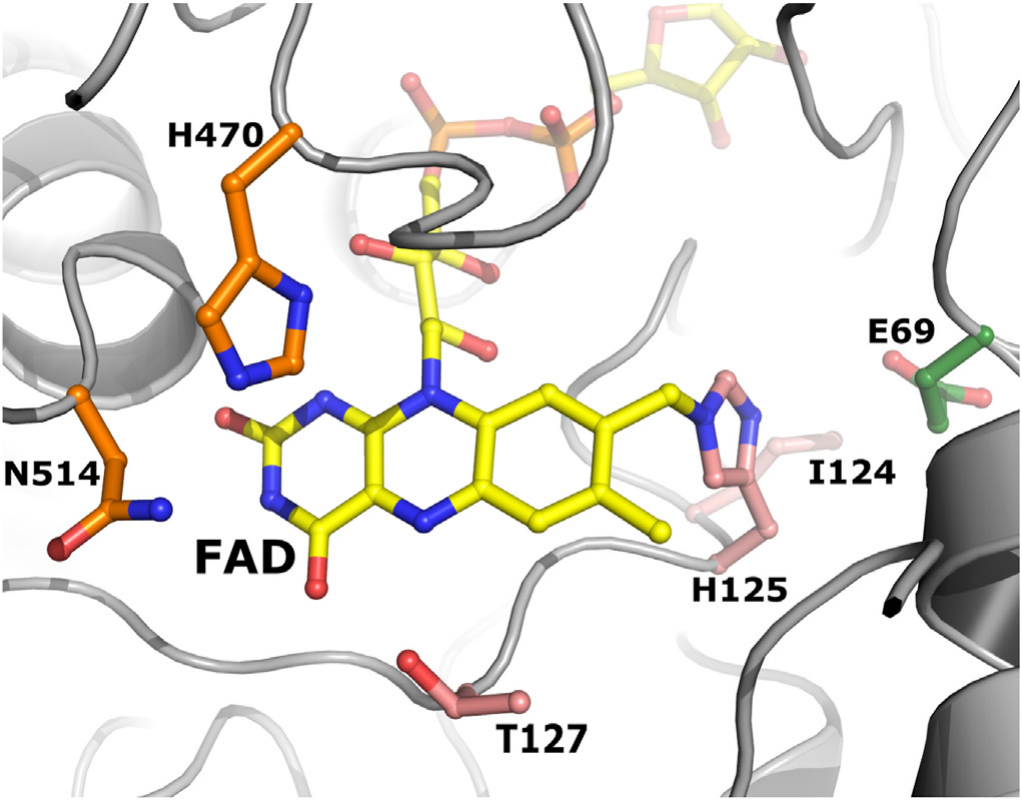
**Close-up view of the active site of *O*POx with its flavin cofactor in yellow.** Catalytic residues are shown as orange sticks, the flavin binding motif is in salmon pink, and the glutamic acid that pushes the His125 toward the flavin is colored forest green.

*O*POx’s monomeric subunit (Fig. 3*B*) displays a typical GMC fold with a Rossmann fold flavin-binding domain and a substrate-binding domain. Similar to other POxs, the substantial central void directs to a narrow substrate channel that is regulated by a substrate loop, which is crystallized in the closed conformation and is hypothesized to rearrange to an (semi-)open state upon substrate proximity. The substrate-loop (^373^DNFSYGIVPDNIDDR^387^) and the flavin binding motif (^123^GIHW^126^) appear to be different from the described clades (23) but are similar in size and retain the conserved His125, Trp126 and the flavin binding motif following Thr127. The conformation of the substrate loop is most similar to that of *To*POx (PDB: 1TT0). The key residues in the substrate recognition loop in *To*POx (17) are conserved in *O*POx as Asp373, Phe375 and Tyr377. The active-site of *O*POx (Fig. 4) bears a close resemblance to other reported POxs structures and in part to the reported G3Ox structure (30). His125-NE2 is indeed covalently bound to the 8α-methyl moiety of FAD as observed in the fungal enzymes. In contrast to G3Oxs, which do not have a covalently attached FAD, the histidine adopts another conformation which is made possible by a glycine residue instead of Glu69 in *O*POx. The FAD is further surrounded by hydrophobic residues and the catalytic residues His470 and Asn514 (the His-Asn pair) (27). For a productive binding mode the sugar must be oriented for oxidation at C2, the substrate-binding loop in the semi-open conformation and the side chain Oγ1 group of Thr127 is pointing away from the flavin N(5)/O(4) locus (45). However, in the determined *O*POx structure the substrate-binding loop is closed and the Oγ1 group of Thr127 is pointing toward O4 and N5 of the FAD (Fig. 4).

### pH optima and spectral switch

**Figure 5:**
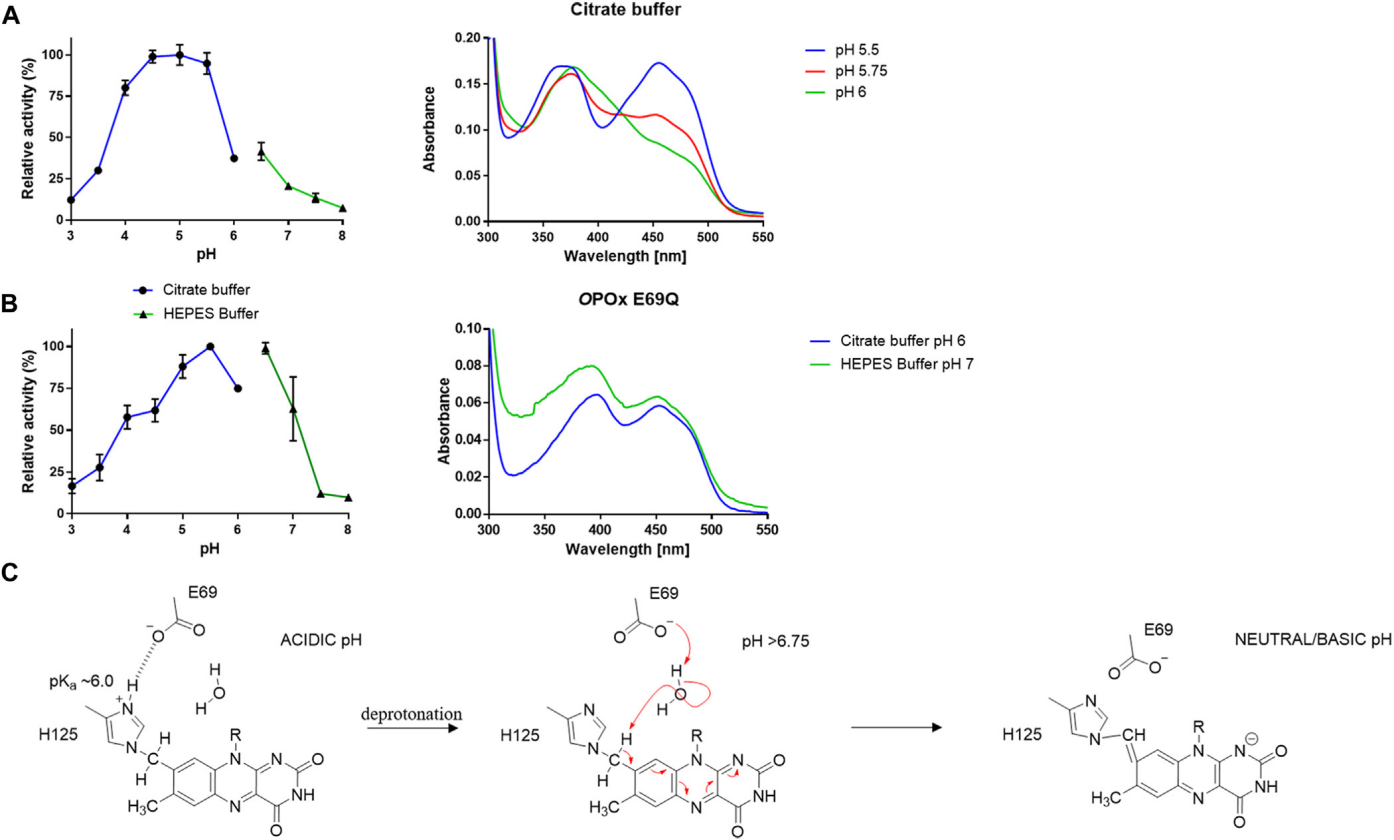
**pH-dependent activity of*****O*****POx.***A*, Left, the effect of pH on the activity of wild-type *O*POx with D-glucose. Right, the absorbance spectra at different pH values. *B*, Left, the effect of pH on the activity of *O*POx E69Q with D-glucose. Right, the absorbance spectra at different pH values. *C*, proposed mechanism of the observed spectral phenomena, wherein a protonated histidine is stabilized by the glutamic acid at acidic pH values and a water-mediated proton transfer “inactivates” the flavin at neutral and basic pH values.

The pH dependency of *O*POx activity shows a narrow optimum at around pH 5 to 5.5, with a sharp decline in activity at high pH value (Fig. 5*A*). As pH optima of activity of other POxs are broader and often favor alkaline conditions as the catalytic residues are reported to be unprotonated for catalytic activity (27), we investigated this atypical behavior. The decline in activity at relatively low pH (< pH 5.5) was also observed for other sequence-related POXs and has been attributed to a relatively low pKa of the histidine in the His/Asn catalytic base (26, 27). This suggests that also in *O*POx, His470 has to be unprotonated in order to act as catalytic base. Intriguingly, we observed a drastic change in the flavin absorbance spectrum when collecting spectra at different pH values (Figs. 5*A* and S2*C*). At relatively high pH, as a result of the disappearance of the absorbance peak at 455 nm, the protein lost it yellow appearance and the flavin absorbance spectrum of *O*POx showed resemblance to a reduced flavin species. By mixing enzyme in 50 mM phosphate buffer (pH 7.5) with 50 mM citrate buffer (pH 4.5), the same spectral change could be observed (Fig. S2*D*). This spectral change was found to be fully reversible, acting as an ‘on-off switch’ around pH 6 to 7. Interestingly, the H125A *O*POx mutant did not display this pH-dependent spectral phenomenon, displaying a typical oxidized flavin spectrum across all investigated pH values. This suggested that the covalent histidyl-flavin linkage plays a role in the pH dependency of activity and flavin absorbance features. A somewhat similar pH-dependent spectral phenomenon has been observed in another GMC oxidoreductase. Choline oxidase from *Arthrobacter globiformis* is known to be purified as a mixture of oxidized and anionic semiquinone reduced species and can be fully ‘rescued’ by incubating the enzyme overnight at pH 6, resulting in fully oxidized enzyme (46, 47). When the covalent bound of the flavin was broken by mutagenesis of the covalent forming histidine, this phenomenon also disappeared and a fully oxidized enzyme was obtained (42). However, the observed spectral phenomenon in *O*POx does not appear to be a semiquinone species as the characteristic absorbance peak and shoulders are missing (48). Choline oxidase is also reported to be able to form adducts between its flavin at the C4a position and nearby active site residues. This is either photoinduced or catalyzed by a buffer component and forms an adduct *via* a cysteine or histidine residue (49, 50). The spectral phenomenon in *O*POx appears to be buffer type independent and investigation of the residues near the C4a of the flavin cofactor in *O*POx shows that they are all highly conserved in other POxs which do not show this spectral switch. Inspection of the crystal structure, which was obtained at a relative high pH (pH 9), also did not show any indication of a flavin adduct. In conclusion, we consider it unlikely that the spectral phenomenon observed in *O*POx can be explained by a mechanism similar to that reported for choline oxidase. Inspection of the crystal structure revealed the presence of a salt-bridge forming glutamic acid (Glu69) near His125 (Fig. 4), which appears to push the histidine into a different conformation than observed in the bacterial POx-like oxidases that contain a non-covalent FAD. Glu69 has an oxygen atom pointing towards the histidine at a distance of 4.4 Å. It may have a role in deprotonating the flavin cofactor which is required for the self-catalytic mechanism of the proposed covalent flavination process (39). At acidic pH values, it is hypothesized that this glutamic acid plays a role in stabilizing the protonated state of the covalently bound forming histidine. At neutral to basic pH values, the histidine is believed to lose its proton, triggering a water-mediated deprotonation of the 8-methyl moiety of the flavin which gives rise to a flavin in a quinone-imine methide state, which is not capable to accept a hydride at N5 (Fig. 5*C*). Such flavin resonance structure would be in line with the observed flavin spectrum and low activity at basic pH. Sequence and structure alignment with POxs known to contain a covalent FAD reveals that *O*POx is the only POx harboring a glutamate at this specific postion (Fig. S1). To further support our hypothesis, a mutant of *O*POx was prepared (E69Q), replacing the glutamic acid with a glutamine, as observed in *To*POx. As predicted, the E69Q *O*POx mutant exhibits a typical oxidized flavin spectrum across all investigated pH values, while still retaining a covalently bound cofactor (Fig. S2*A*). Furthermore, the mutant is obtained mainly in its holo form evidence by a ratio of 11.3 in absorbance at 280 nm and 455 nm and seems to retain activity at higher pH values (Fig. 5*B*). The pH optimum for activity changed only to a small extent, hinting that more residues involved that dictate the pH optimum for activity. The E69Q mutant resulted in a 7-fold decrease in catalytic efficiency for D-glucose (7 mM^-1^ s^-1^), mainly due to its increased *K*_*M*_ value of 225 μM (Fig. S5*I*).

## Discussion

In this study, a novel bacterial POx from *O. princeps*, dubbed *O*POx was discovered, biochemically characterized and its structure was elucidated at a resolution of 2.6 Å. It marks the first reported bacterial POx crystal structure. Structurally, *O*POx is highly similar to fungal POxs as it is a homotetramer in solution with a big central void leading to a narrow substrate channel. This channel leads to the active site where the flavin cofactor is covalently bound to a histidine. Interestingly, the head domain of *O*POx is significantly smaller while it still remains a tetrameric formation, downplaying its role in oligomerization. The arm domain seems to play a dominant role in formation of tetramers. *O*POx is endowed with favorable biocatalytic characteristic as it is the most thermostable POx described so far with a melting temperature exceeding 90 °C and it can be functionally expressed in *E. coli*. Interestingly, it displays an extremely low *K*_*M*_ for D-glucose which is, to our knowledge, the lowest *K*_*M*_ value described in literature for D-glucose for an oxidase. This makes the enzyme a prime target for use in biotechnological applications such as in biosensors and in synthetic chemistry as biocatalyst.

Intriguingly, *O*POx shows a rather narrow pH optimum for activity. This is in a large part due to the low activity at pH > 6 caused by a unique mechanism of flavin inactivation. At higher pH values, the flavin switches to a redox-inactive state. The role of glutamic acid (Glu69) in this process was confirmed by site-directed mutagenesis. The proposed mechanism of flavin inactivation at higher pH values involves a change in the protonation state of the histidine that is tethered to the flavin, resulting in a resonance from the flavin cofactor which is not capable of accepting a hydride from a substrate (Fig. 5*C*). This mechanism seems unique for this bacterial pyranose oxidase when considering other known flavoproteins. All-in-all, *O*POx presents itself as a valuable new biocatalytic tool for biotechnological applications with a unique pH-dependent “on-off” switch.

## Experimental procedures

### Chemicals and materials

Chemicals were purchased from Sigma-Aldrich. HiLoad Superdex 200 16/60 column for size-exclusion chromatography and Ni-Sepharose 6 Fast Flow for protein purification were obtained from Cytiva.

### Genome mining

Putative bacterial POx sequences were identified by utilizing the PSI-BLAST tool of the National Center for Biotechnology Information, with default setting and the sequence of POx from *T. ochracea* (GenBank: 1TT0_A) as search query. Putative POx sequences were compared by aligning them with known POxs using ESpript (51) and structures of promising sequences were predicted using the online AlphaFold tool ColabFold (24, 29). As a final selection, structures were aligned with the crystal structure of POx from *T. ochracea* (PDB: 1TT0) using PyMols alignment function.

### Cloning, transformation, and mutagenesis

The synthetic gene of *O*POx (GenBank: MBW4497712) was codon optimized for *E.coli* using Twist Biosciences build-in tools. The synthetic gene was ordered from the same company with Bsa1 sites at its 5′and 3′-termini. Using the Golden Gate methodology, the gene was cloned into a pBAD vector with a N-terminal His-tag (52). For transformation a heat-shock protocol was followed, where 4 μl of PCR product was mixed with 40 μl of *E. coli* NEB 10-beta CaCl_2_ competent cells and kept on ice for 30 min. After which the cells were heat-shocked for 45 s at 42 °C and cooled down again for 5 min on ice. After an hour recovery time at 37 °C in 250 μl LB the cells were plated on LB agar supplemented with 50 μg/ml ampicillin (amp) and left overnight at 37 °C. Obtained colonies were picked and grown in 5 ml LB with 50 μg/ml amp overnight at 37 °C. After which plasmid isolation could be performed and cloning was verified using sequencing. Primers for mutagenesis were ordered from Sigma-Aldrich and are shown in Table S3. Mutagenesis was done using the QuickChange methodology (53). PCR mixture consisted out of 100 ng template plasmid, 1 μM of both reverse and forward primer, 2% DMSO, 0.8 μM MgCl_2_, 12.5 μl PfuUltra II Hotstart PCR Master mix and filled to a final volume of 25 μl with MiliQ water.

### Protein expression, purification, and characterization

For expression purposes, an overnight culture (37 °C, 200 rpm) of *O*POx in 5 ml LB containing 50 μg/ml amp was resuspended into 500 ml of Terrific Broth medium supplemented with 50 μg/ml amp and incubated at 37 °C, 200 rpm in a baffled flask. After an OD_600_ of ∼0.6 was reached, the culture was induced by adding a final concentration of 0.02% L-arabinose and incubated overnight (∼16 h) at 24 °C, 200 rpm. Cells were harvested by centrifuging at 3700 rpm, 4 °C for 20 min and pellets were stored at −22 °C.

For purification, cell pellets were resuspended into 50 ml lysis buffer (50 mM TRIS-HCl, 150 mM NaCl, pH 8) with 0.1 μM phenylmethanesulfonyl fluoride and cells were disrupted by sonication (5s on, 7s off, 70% amplitude for 12 min) in an ice bath. Supernatant was harvested by centrifuging at 11.000 rpm, 4 °C for 50 min and was loaded onto a 2 ml lysis buffer equilibrated Ni-Sepharose containing column. The column was washed three times with a column volume of lysis buffer and three times with a column volume of wash buffer (50 mM TRIS-HCl, 150 mM NaCl, 20 mM imidazole, pH 8). *O*POx was eluted off the column with 3.5 ml of elution buffer (50 mM TRIS-HCl, 150 mM NaCl, 500 mM imidazole, pH 8). After which the elution buffer was exchanged for 3.5 ml 50 mM citrate buffer (pH 5.5) with a PD10 desalting column. The enzyme sample was heated in a water bath at 70 °C for 1 h. *O*POx was obtained in the supernatant after centrifuging at 11.000 rpm, , 4 °C for 10 min and stored at 4 °C.

The molar extinction coefficient of *O*POx at 455 nm was determined by comparing its spectra before and after denaturation with 0.1% SDS using a JASCO V-660 spectrophotometer. The found ε_455_ was used to determine enzyme concentrations.

The oligomeric state in solution was studied with dynamic light scattering (DLS). Where 6 μl of *O*POx (∼15 μM) in 50 mM citrate buffer (pH 5.5) was centrifuged at 10.000 rpm and measured with DynaPro NanoStar MS800 TC instrument (Wyatt Technology Corporation) at 294 K. The resulting data were analyzed using the DYNAMICS software.

All mass photometry experiments were performed utilizing the Refeyn (TwoMP mass photometry setup, with data capture facilitated by the AcquireMP software. Microscope coverslips measuring 24 mm × 50 mm (VMR) underwent a rigorous cleaning method, beginning with immersion in a 3% mucasol solution for 30 min. Subsequently, the coverslips underwent thorough washing step including rinses in HPLC-grade ethanol and Milli-Q H_2_O, each repetition consisting of at least three cycles. Following this, the coverslips were meticulously dried using compressed air. For experimental setup, a 6-well silicon gasket was fixed onto a clean coverslip and inserted into the instrumentation. Protein samples were appropriately diluted to attain a working concentration within the nanomolar range. The measurement protocol involved the addition of 10 μl of buffer, filtered with 0.22-μm filter, into the designated well. The focal point was determined using the droplet dilution feature of the AcquireMP software. Upon achieving focus, 10 μl of the protein sample was gently introduced into the same well and thoroughly mixed. Subsequently, data collection was started and carried out for 1 minute following the mixing event. Data processing and analysis were carried out using the DiscoverMP software, also from Refeyn.

The melting temperatures of *O*POx were determined using the ThermoFluor assay in triplicate (32). The assay was performed from 20 °C to 99 °C with an increase of 1 °C every 1 min, by diluting 150 μM *O*POx stock (50 mM citrate buffer, pH 5.5) 10-fold in different buffers, ranging from 4 pH to 8 pH with and without common cosolvents (10%). The half-life of *O*POx was determined at 90 °C by incubating 200 μl of *O*POx in a 90 °C water bath. A regular time intervals, samples of 10 μl were taken to measure activity at 25 °C with 1.0 mM D-glucose using an horse radish peroxidase (HRP)/4-aminoantipyrine (AAP)/3,5-dichloro-2-hydroxybenzenesulfonate (DCHBS) assay JASCO V-660 spectrophotometer (34).

pH optima of *O*POx were determined by measuring activity against 1.0 mM D-glucose in an Oxygraph plus system (Hansatech Instruments Ltd) using different pH buffers (50 mM solutions of citrate, potassium phosphate or HEPES buffers), in duplicates. D-glucose solutions were prepared in appropriate buffers and reactions were initiated by adding 1:10 *O*POx in 50 mM citrate buffer (pH 5.5).

For collecting spectra at different pH values, enzyme samples were prepared using a PD10 buffer exchange column equilibrated with the desired buffer. Absorbance spectra were obtained using a JACSO V-660 spectrophotometer. When comparing spectra, enzyme concentrations were normalized based on their 280 nm absorbance while using the same enzyme stock solution and protocol for changing buffer.

### Product determination

A 10 ml reaction between 3 μM *O*POx and 10 mM D-glucose in 50 mM acetate buffer (pH 5.5) was incubated for 24 h at 24 °C, 135 rpm. Water was evaporated by flash freezing samples in liquid nitrogen and freeze drying them overnight (∼16 h). Obtained powder was dissolved in 500 μl DMSO-d6 and analyzed through ^1^H COSY NMR in a Bruker Avance NEO 600, 600 MHz. Spectra’s were analyzed using Mnova V.15 software.

### Redox potential

Redox potentials were determined using the xanthine/xanthine oxidase method (40, 41). Where 20 μM of enzyme in 50 mM citrate buffer (pH 5.5) with a catalytic amount of xanthine oxidase, 300 μM xanthine, 5 μg/ml catalase, 20 μM benzylviologen, 10 mM xylitol and 1.0 μM of alditol oxidase from *Thermopolyspora flexuosa* V258L P259I (54) were mixed with suitable dye and followed in a 1 ml quartz cuvette using a JASCO V-660 spectrophotometer at 25 °C. Anaerobic conditions were assured by purging the curvet with argon for 15 min and removing remaining oxygen by the oxidation of xylitol by the alditol oxidase from *T. flexuosa* V258L P259I (54). After the anaerobic condition were met, the reaction was initiated by the addition of the xanthine oxidase and spectra’s were recorded for an hour. Indigotetrasulfonate (E_0_ = −46 mV) was found as a suitable dye and the redox potential was determined by using its appropriate Nernst equation (40, 41).

### Steady state kinetics

Steady-state parameters were determined using an HRP/AAP/DCHBS assay in 50 mM citrate (pH 5.5) buffer at 25 °C with a JASCO V-660 spectrophotometer (34). The mixture consisted of 0.10 μM enzyme, 0.10 mM AAP, 1.0 mM DCHBS, 0.013 mg/ml HRP and varying substrate concentrations. Appropriate substrate concentrations were found by initially screening 1.0 mM substrate and increasing or decreasing the concentration to obtained an estimate of the K_*M*_ value. Based on that, substrate ranges were used that allow measurements of rate that are well above and below the K_*M*_ value. Reactions were initiated by adding enzyme and initial linear increase of color formation (515 nm) was used to determine *k*_*obs*_ values by using the molar extinction coefficient (26 mM^-1^ cm^-1^) of the resulting colored product (34). Data was analyzed using a Michaelis-Menten curve model with the GraphPad Prism 8.0 software.

The *K*_*M*_ for oxygen of *O*POx was probed by monitoring the depletion of molecular oxygen in duplicate under atmospheric pressure (240 μM) in 50 mM citrate (pH 5.5) solution by the reaction between 0.1 μM *O*POx and an excess of D-glucose (10 mM) using an Oxygraph plus system (Hansatech Instruments Ltd) (60 rpm, 25 °C). The progress curve analyses was performed by determining the *k*_*obs*_ values for every step of 25 μM oxygen consumption. The obtained *k*_*obs*_ values were used for fitting with a Michaelis-Menten model using GraphPad Prism 8.0 software.

### Pre-steady-state kinetics

The reductive half-reaction of *O*POx was monitored at 25 °C in a nitrogen-purged anaerobic chamber of a S20X stopped-flow instrument (Applied Photophysics). To ensure anaerobic conditions of enzyme and substrate solution, 10 mM xylitol and 1 μM alditol oxidase from *T. flexuosa* V258L P259I (54) were added after a nitrogen purge of 10 min. The reduction of 6 μM *O*POx was monitored in duplicates with different glucose concentrations ranging from 30 μM to 1 mM. Spectral scans were taken every millisecond and data was analyzed using Pro-Kinetics V1.0.13 and reduction rates were subsequently determined using GraphPad Prism 8.0. The oxidative half-life was followed using the same set-up at 25 °C where D-glucose reduced *O*POx was re-oxidized with aerobic 50 mM citrate buffer (pH 5.5). D-Glucose reduced *O*POx was prepared by mixing 8 μM *O*POx with 20 μM D-glucose under anaerobic conditions. Mixing the two solutions in the stopped flow resulted in an oxygen concentration of ∼120 μM. Spectral scans were taken every millisecond and data was analyzed in a similar fashion as the reductive half-reaction.

### Protein crystallization and structure elucidation

For crystallization; *O*POx was further purified by gel permeation using a Superdex 200 HR10/30 column (Cytiva), equilibrated with 20 mM Acetate pH 5.3, 150 mM NaCl on an Äkta explorer system (Cytiva) with wavelengths set at 280, 254 and 460 nm. Light yellow-colored *O*POx fractions were pooled and concentrated to 5.9 mg ml^-1^ using an Ultracel-30K filter unit (Millipore).

Initial sitting-drop crystallization screening was performed using a Mosquito crystallization robot (STP Labtech) in 96-well MRC2 plates (Swissci). Small crystals were grown in the PACT screen with 0.1 M bis-tris propane buffer (pH 8.5) and 20% PEG3350 supplied with 0.2 M sodium nitrate, sulfate, citrate, or malonate. Prior to data collection, crystals were briefly soaked in a cryoprotectant containing the crystallization solution supported with 25% glycerol and flash-cooled in liquid nitrogen. X-ray diffraction data were recorded at the MASSIF-1 beamline at the ESRF, Grenoble (55).

Automatic data processing, using the program autoPROC with anisotropic analysis (56), was performed at the ESRF. A low-resolution crystal could be grown with sodium nitrate as an additive at pH 8.5 in space group *C*222_1_ with cell dimensions a = 74.7, b = 150.1 and c = 213.4 Å. This crystal form contained a dimer in the asymmetric unit, tetramer formation is through a two-fold crystallographic symmetry axis, but was not considered for further research. More suitable crystals, diffracting to 2.64 Å obtained with sodium sulfate as additive at pH 9.2, belonged to space group *P*2_1_2_1_2_1_ with cell dimensions of a = 106.0, b = 137.4 and c = 143.6 Å. The V_M_ is 2.2 Å^3^/Da (57) with a solvent content of 44%. The structure of *O*POx could be determined by molecular replacement using Phaser (58) with an Alphafold2 (ColabFold) model. The asymmetric unit contained four monomers of 61.0 kDa. Refinement and model building, was done using the programs Coot (59) and REFMAC5 using NCS and TLS (60). The FAD cofactor was built in the Fo – Fc electron density.

The quality of the model was analyzed with PDB_REDO (61) and MolProbity (62). PyMOL (Schrödinger) was used for figure preparation. Data collection statistics and refinement details are shown in Table S4. Atomic coordinates and experimental structure factor amplitudes were deposited in the Protein Data Bank PDB number 9FL2.

## Data availability

This article contains supporting information. All data generated and analyzed in this study is included in this manuscript and its supplementary information. The obtained structure is available in the protein databank (PDB 9FL2).

## Supporting information

This article contains supporting information.

## Conflict of interest

The authors declare that they have no conflicts of interest with the contents of this article.
