# Supplementary material for: Identification of a robust bacterial pyranose oxidase that displays an unusual pH dependence
Source: J Biol Chem. 2024 Oct 11;300(11):107885. doi: 10.1016/j.jbc.2024.107885 (PMC11650716; doi:10.1016/j.jbc.2024.107885)
Supplement: Supporting information [file mmc1.docx]

**Supplementary information**

**Identification of a robust bacterial pyranose oxidase which displays an unusual pH dependence**

Lars L. Santema^[a]^, Henriëtte J. Rozeboom^[a]^, Veronica P. Borger^[a]^, Saniye G. Kaya^[a]^, Marco W. Fraaije^[a]*^

^[a]^ Molecular Enzymology, University of Groningen, Nijenborgh 3, 9747AG Groningen, The Netherlands

^*^ Corresponding author: [m.w.fraaije@rug.nl](mailto:m.w.fraaije@rug.nl)

**This additional file contains:**

**Tables:** S1, S2, S3, S4 (pages 2 & 3)

**Figures:** S1, S2, S3, S4, S5, S6, S7 (pages 4-10)

***Table S1:*** bacterial family used as search databases for pyranose oxidase homologues

| **Genus** |
| --- |
| *Pyrococcus* |
| *Phormidium* |
| *Oscillatoria* |
| *Bacillus* |
| *Thermus* |
| *Thermoanaerobacter* |
| *Thermotogae* |

***Table S2:*** Substrates tested for activity with *O*POx using a HRP/AAP/DCHBS method (32). All substrate concentrations were 10 mM and the reaction was performed in 50 mM citrate buffer (pH 5.5). X means no observed activity and V means activity was observed.

| Substrate (10mM) | Activity |
| --- | --- |
| *Monosaccharides* | |
| D-Glucose | V |
| D-Galactose | V |
| D-Mannose | V |
| D-Fructose | V |
| D-xylose | V |
| L-arabinose | V |
| N-acetyl Glucosamine | V |
| N-acetyl Galactosamine | X |
| N-acetyl Mannosamine | X |
| Rhamnose | V |
| D-ribose | V |
| L-fucose | V |
| D-lyxose | V |
| *Sugar alcohols* | |
| Xylitol | X |
| Ribitol | X |
| Sorbitol | X |
| D-arabitol | X |
| Galactitol | X |
| Vanillyl alcohol | X |
| *Aromatic alcohols* | |
| Ferulic acid | X |
| Caffeic acid | X |
| Sinapic acid | X |
| Coumaric acid | X |
| D-galacturonic acid | V |
| Syringic acid | X |
| *Glycosylated saccharides* | |
| Naringin | X |
| Carminic acid | X |

***Table S3:*** Primers used for mutagenesis

| **Primer** | **Sequence 3’-‘5** |
| --- | --- |
| H125A Fw | GTATGGGGATA**GCG**TGGACTTGTGCCAC |
| H125A Rv | CAAGTCCA**CGC**TATCCCCATACCGCCTAC |
| E69Q Fw | GTCAATTCG**C**AGCTTTACAAGTTGAG |
| E69Q Rv | GTAAAGCT**G**CGAATTGACGATTTG |

***Table S4.*** Crystallographic data collection and refinement statistics. Values in parentheses are for the highest resolution shell.

| **Data crystal *O*POX** | |
| --- | --- |
| Resolution range (Å) | 99.3 – 2.64 (2.87 -2.64) |
| Cell dimensions (Å) *a ,b, c* | 106.0, 137.4, 143.6 |
| Number of observations | 489727 (20361) |
| Number of unique reflections | 38297 (1915) |
| Completeness (spherical)(%) | 61.5 (14.2) |
| Completeness (ellipsoidal)(%) | 88.6 (63.7) |
| Multiplicity | 12.8 (10.6) |
| CC_1/2_ | 0.941 (0.296) |
| Overall I/σ (I) | 7.3 (1.5) |
| *R_merge_* (%) | 0.364 (2.047) |
| *R_pim_* (%) | 0.106 (0.727) |
| *R/ Rfree* (%) | 18.7 / 25.0 |
| Protein residues | 2107 |
| Protein *B* value (Å^2^) | 42.6 |
| FAD molecules | 4 |
| FAD *B* values (Å^2^) | 35.8 |
| Chloride ions | 3 |
| Water molecules | 73 |
| **Validation** | |
| r.m.s.d. bonds (Å) | 0.006 |
| r.m.s.d. angles (°) | 1.62 |
| Ramachandran outliers | 0.1 |
| favored | 94.8 |
| Clashscore | 6.5 |
| Molprobity score | 1.90 |
| PDB accession ID | 9FL2 |


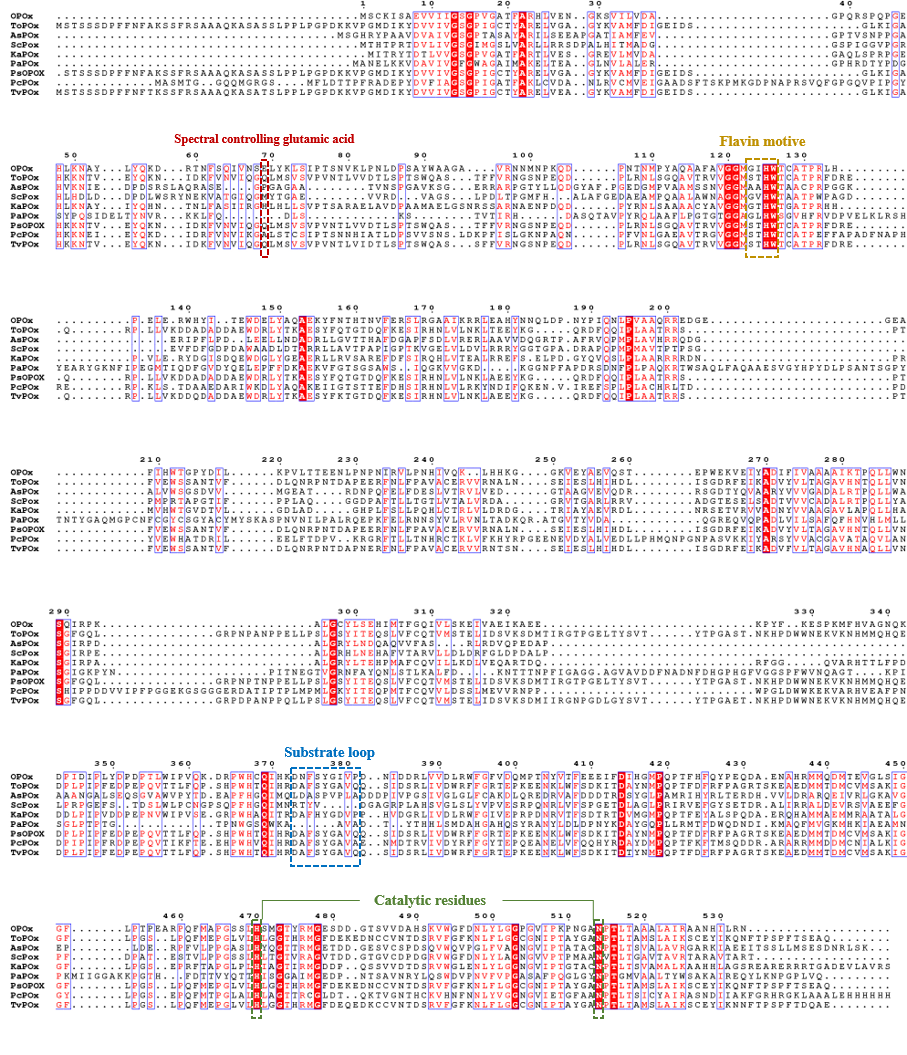
 **Figure S1:** Multiple sequence alignment of *O*POx and thus far described POxs generated with ESpript (51). Conserved residues are highlighted with a red background. The FAD-motive is surrounding by a dotted yellow line, the substrate loop by a dotted blue line, the catalytic residues by a dotted green line and the spectral phenomenal controlling glutamic acid by a dark red dotted line. Positions and residues, besides spectral phenomenal controlling glutamic acid the spectral, are based on Kostelac et al (23).


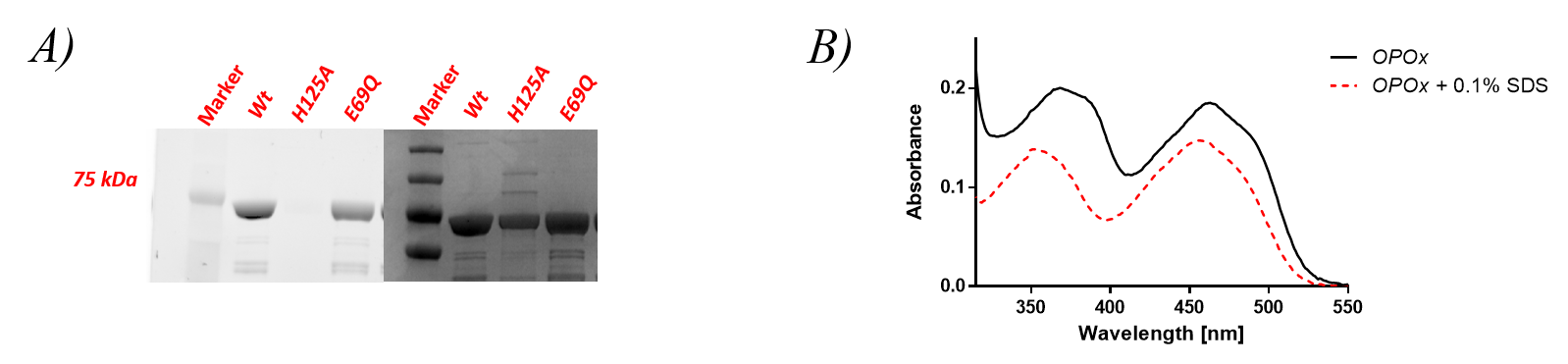
**A**


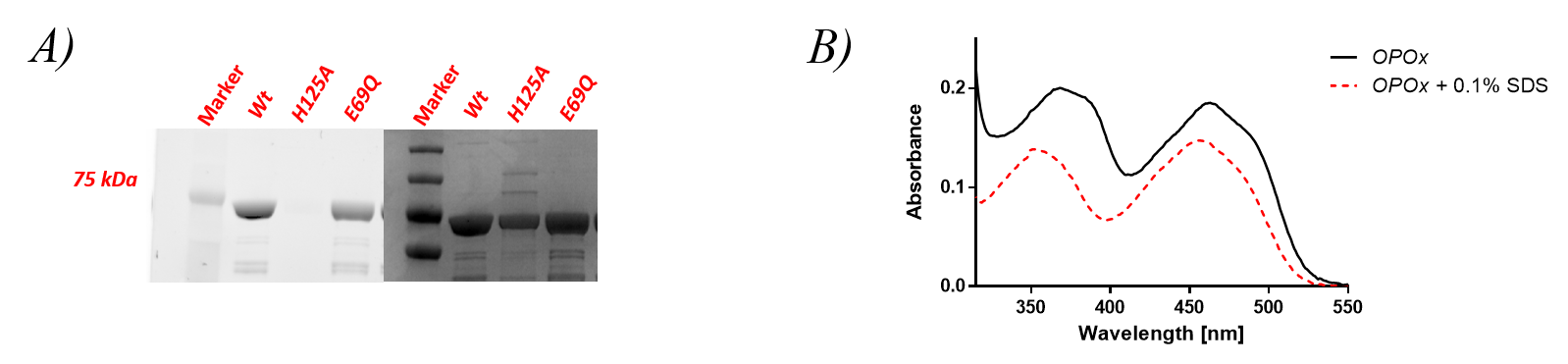


**B**

**
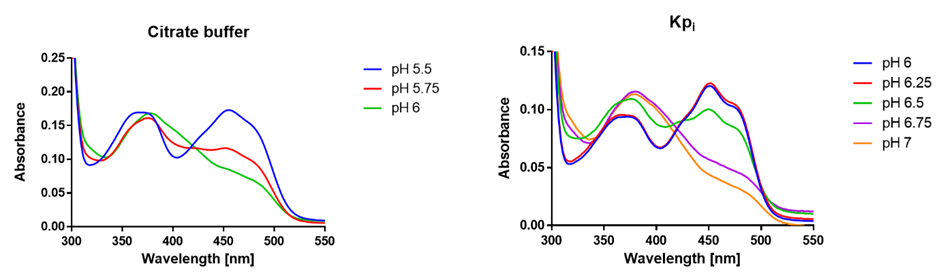
**

**C**


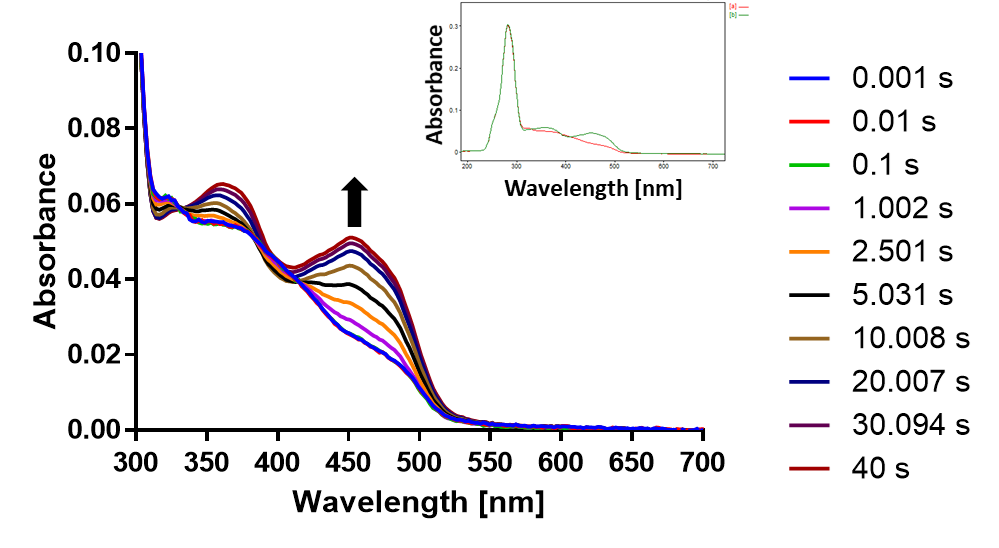


**D**

**Figure S2:** **A)** SDS-PAGE gel (12%) of His-tagged and heat purified *O*POx and its mutants. The right image shows the Coomassie stained gel and the left one shows the same gel under UV-light after being incubated with 5% (v/v) acetic acid. *Wt* for the *wild-type O*POx, H125A for the mutant with the lacking flavin-protein linkage and E69Q for the glutamic acid mutant. **B)** UV-Vis absorbance spectra of *O*POx, before (solid black line) and after treatment with 0.1% SDS (broken red line), in 50 mM citrate buffer pH 5.5 (25 °C). **C**) Absorbance spectra of *O*POx collected using phosphate buffers at different pH values. D) Absorbance spectra obtained by stopped-flow upon mixing 10 µM *O*POx in 50 mM phosphate (pH 7.5) with 50 mM citrate buffer (pH 4.5), resulting in a final pH of 5.5. Spectra were collected at the indicates time intervals and revealed a process with a rate of 0.14 s^-1^ (the inset shows the deconvoluted spectra).


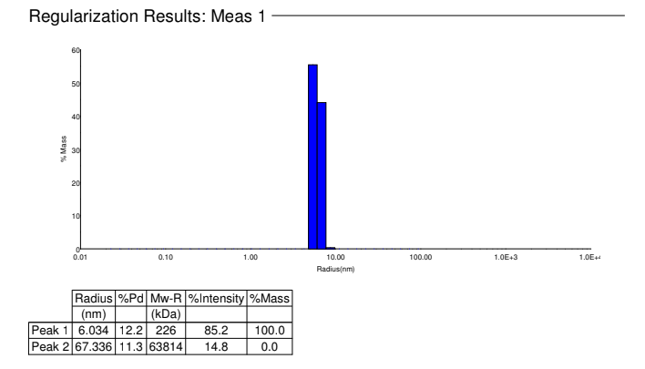


**Figure S3:** Resulting analysis from dynamic light scattering of *O*POx. The table indicates the predicted mass.


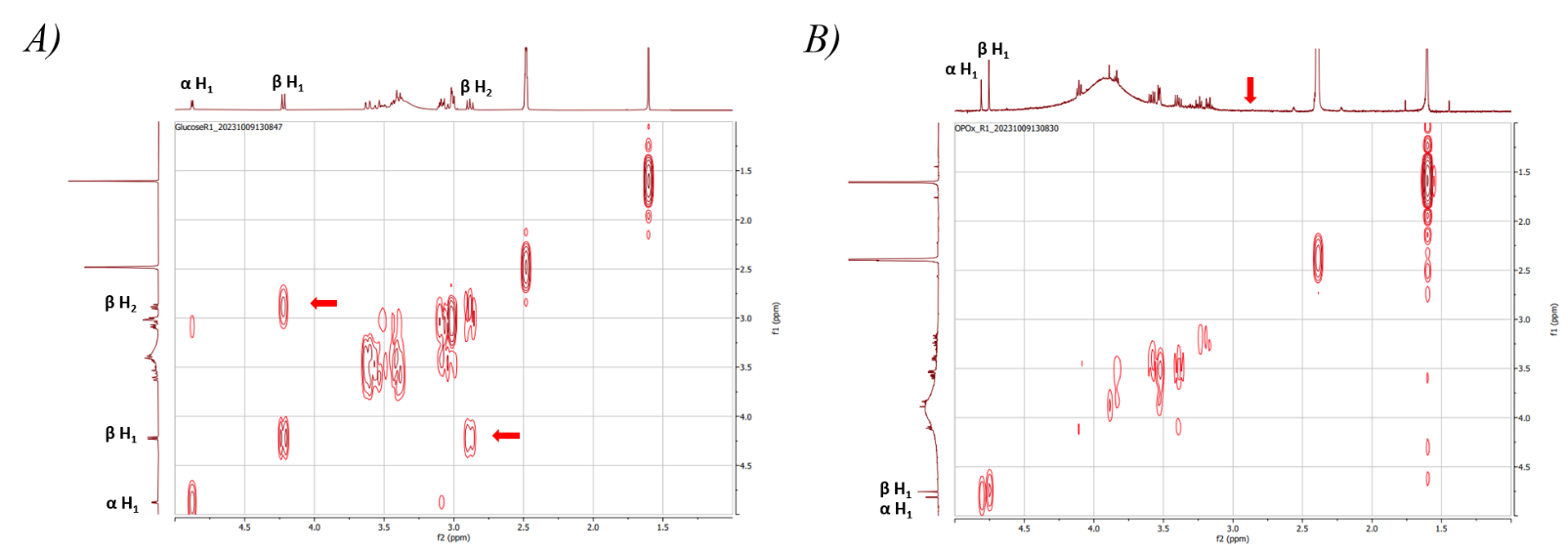
**Figure S4:** 2D-COSY NMR spectra of D-glucose (**A**) and the oxidation product of the *O*POx reaction with D-glucose (**B**). The red arrows indicate the peaks that are absent in the product, indicating the formation of 2-keto-glucose. The reaction was performed in 50 mM acetate buffer (pH 5.5) at 24 °C for 24 hours and consisted out of 10 mM of D-glucose and 3.0 µM *O*POx.


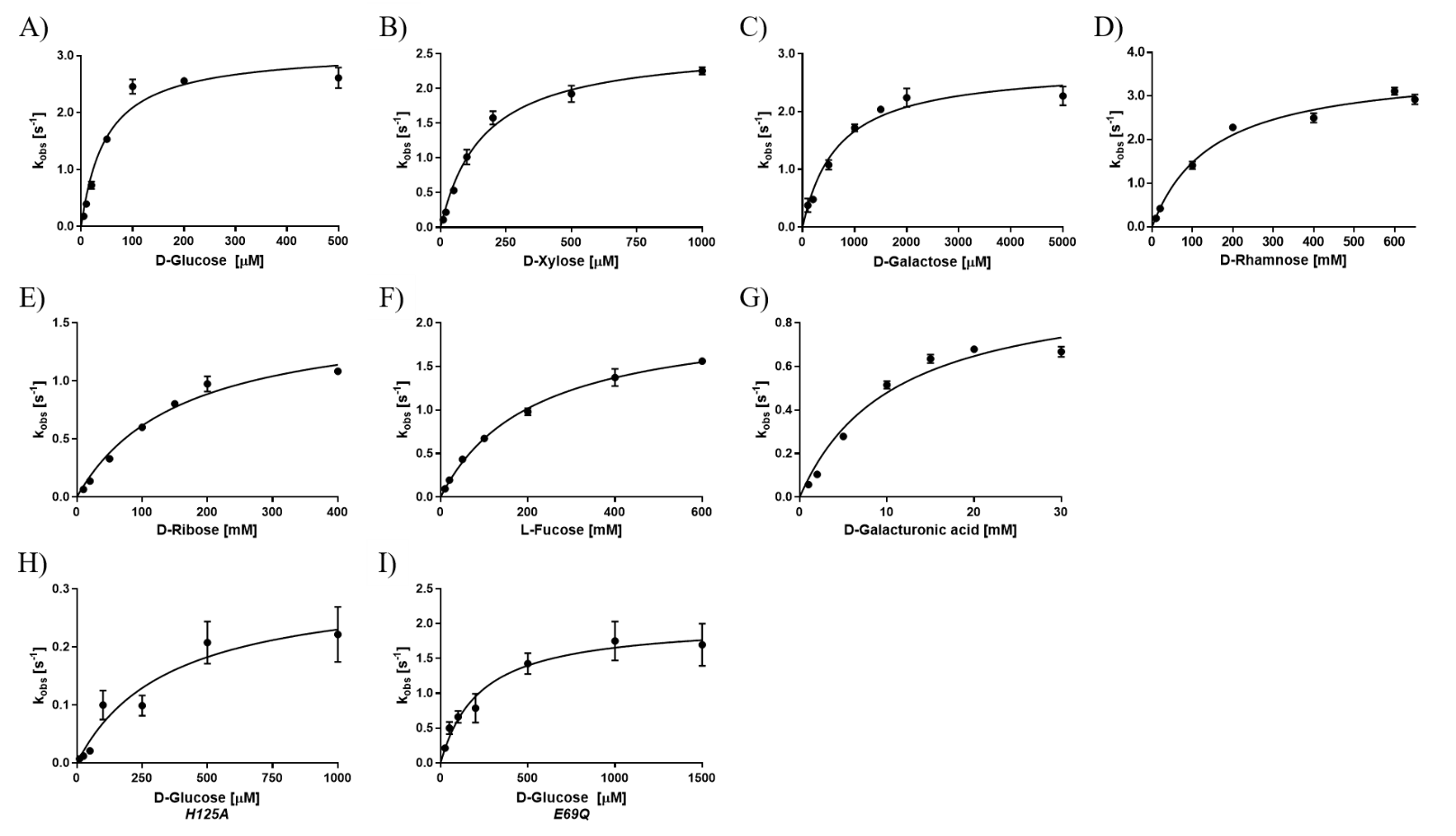


**Figure S5:** Michaelis-Menten fitted curves of *O*POx with tested carbohydrates: **A)** D-glucose, **B)** D-xylose, **C)** D-galactose, **D)** D-rhamnose, **E)** D-ribose, **F)** L-fucose and **G)** D-galacturonic acid. **H)** *O*POx H125A with D-glucose. **I)** *O*POx E69Q with D-glucose. All experiments where performed in 50 mM citrate buffer (pH 5.5) at 25 ⁰C. Error bars indicate SD and all point were taken in triplicates (*n* = 3).

**Figure S6:** *O*POx activity with an excess of D-glucose (1.0 mM) at various oxygen concentrations in 50 mM citrate buffer (pH 5.5). The experiments were performed in 50 mM citrate buffer (pH 5.5) at 25 ⁰C. Error bars indicate SD and all point were taken in triplicates (*n* = 3).


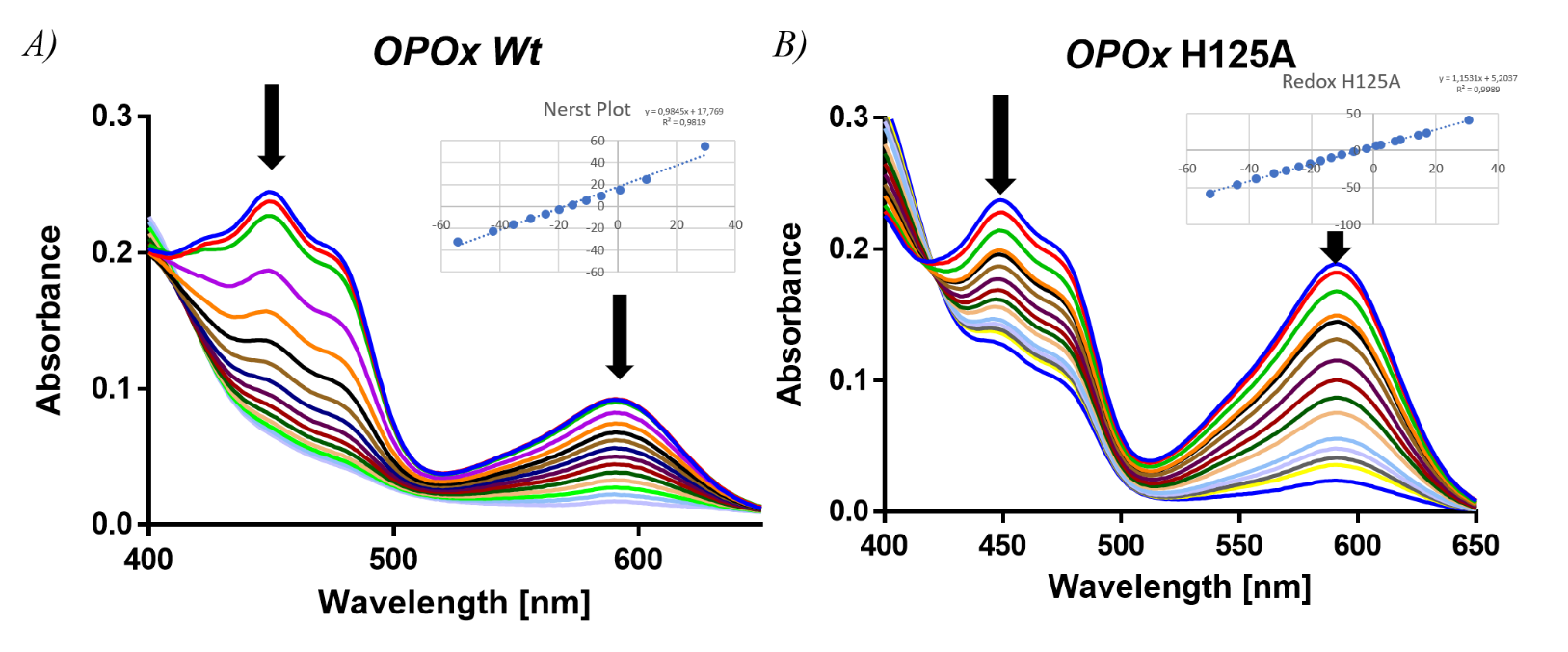


**Figure S7:** Redox potential of *O*POx (**A**) and *O*POx H125A (**B**) found with xanthine/xanthine oxidase methode and indigotetrasulfonate as a dye. The insets shown the derivate Nerst plots (x/y: Log[Ox_dye_/Red_dye_]/Log[Ox_enzyme_/Red_enzyme_]).
